# Supplementary material for: Cell lineage and cell cycling analyses of the 4d micromere using live imaging in the marine annelid Platynereis dumerilii
Source: eLife. 2017 Dec 12;6:e30463. doi: 10.7554/eLife.30463 (PMC5764573; doi:10.7554/eLife.30463)
Supplement: Transparent reporting form [file elife-30463-transrepform.pdf]

1st Floor
24 Hills Road
Cambridge CB2 1JP, UK
P 01223 855340
W elifesciences.org
T @elife

eLife's transparent reporting form

We encourage authors to provide detailed information *within their submission* to facilitate the interpretation and replication of experiments. Authors can upload supporting documentation to indicate the use of appropriate reporting guidelines for health-related research (see EQUATOR Network), life science research (see the BioSharing Information Resource), or the ARRIVE guidelines for reporting work involving animal research. Where applicable, authors should refer to any relevant reporting standards documents in this form.

If you have any questions, please consult our Journal Policies and/or contact us: editorial@elifesciences.org.

Sample-size estimation

- You should state whether an appropriate sample size was computed when the study was being designed
- You should state the statistical method of sample size computation and any required assumptions
- If no explicit power analysis was used, you should describe how you decided what sample (replicate) size (number) to use

Please outline where this information can be found within the submission (e.g., sections or figure legends), or explain why this information doesn't apply to your submission:

Our work concerns a descriptive analysis of cell lineage and cell cycling patterns using advanced live imaging. Injections and live imaging was carried out repeatedly with success over the course of 2 years. Criteria for selecting healthy embryos and larvae to be imaged can be found in Methods under "Mounting Samples for Imaging" on pages 32 and 33. Cell lineage analysis carried out was curated manually by BDO, and because this not a software-automated task, the authors decided confirming lineages and cell cycle patterns in 3 different samples will support the conclusions with fair confidence. The data from these 3 different samples are reported across the manuscript.

Replicates

- You should report how often each experiment was performed
- You should include a definition of biological versus technical replication
- The data obtained should be provided and sufficient information should be provided to indicate the number of independent biological and/or technical replicates
- If you encountered any outliers, you should describe how these were handled
- Criteria for exclusion/inclusion of data should be clearly stated
- High-throughput sequence data should be uploaded before submission, with a private link for reviewers provided (these are available from both GEO and ArrayExpress)

Please outline where this information can be found within the submission (e.g., sections or figure legends), or explain why this information doesn't apply to your submission:

eLIFE
1st Floor
24 Hills Road
Cambridge CB2 1JP, UK
P 01223 855340
W elifesciences.org
T @elife

All the analyses and experiments reported in this work were based on at least n=3 samples unless stated othewise (in which case, more than (and never less than) 3 samples were analyzed). Specifically, we report the following information in the Results section:
- Page 8 - Pdu-FUCCI injection and analysis (Figure 1B): one representative embryo was shown from n=10, 4 replicates.
- Page 9 - EdU+injection experiment (Figure 1C-D’’’): hundreds of cell nuclei from 6 embryos (from 2 independent experiments) were reported.
- Page 11 – Immunohistochemistry+EdU experiment (Figure 2D-G’): More than 100s of embryos were treated, raised, fixed, immunostained; n=4-6 of representative samples for each batch were imaged, one representative larva is shown in Figure 2
- Page 11-12 – EdU for putative Primordial Germ Cells (Figure 3A-D): sample numbers are detailed in text as well as in Figure 3 legend.
- For live imaging, lineage tracing, cell cycle results reported through Pages 14-20 (remaining figures), one sample named “Sample A” was shown in the majority of the figures. However these results were replicated in 2 more samples (Samples B, and C) and information is provided as supplementary material (or figure supplements), and details are in the Methods section.

Statistical reporting

- Statistical analysis methods should be described and justified
- Raw data should be presented in figures whenever informative to do so (typically when N per group is less than 10)
- For each experiment, you should identify the statistical tests used, exact values of N, definitions of center, methods of multiple test correction, and dispersion and precision measures (e.g., mean, median, SD, SEM, confidence intervals; and, for the major substantive results, a measure of effect size (e.g., Pearson's r, Cohen's d)
- Report exact p-values wherever possible alongside the summary statistics and 95% confidence intervals. These should be reported for all key questions and not only when the p-value is less than 0.05.

Please outline where this information can be found within the submission (e.g., sections or figure legends), or explain why this information doesn't apply to your submission:

Our work concerns a descriptive analysis of cell lineage and cell cycling patterns using advanced live-imaging. Thus, other than replicating samples, we did not employ any statistical analyses.

(For large datasets, or papers with a very large number of statistical tests, you may upload a single table file with tests, Ns, etc., with reference to sections in the manuscript.)

Group allocation

- Indicate how samples were allocated into experimental groups (in the case of clinical studies, please specify allocation to treatment method); if randomization was used, please also state if restricted randomization was applied
- Indicate if masking was used during group allocation, data collection and/or data analysis

Please outline where this information can be found within the submission (e.g., sections or figure legends), or explain why this information doesn't apply to your submission:

Not applicable

Additional data files ("source data")

- We encourage you to upload relevant additional data files, such as numerical data that are represented as a graph in a figure, or as a summary table
- Where provided, these should be in the most useful format, and they can be uploaded as "Source data" files linked to a main figure or table
- Include model definition files including the full list of parameters used
- Include code used for data analysis (e.g., R, MatLab)
- Avoid stating that data files are "available upon request"

Please indicate the figures or tables for which source data files have been provided:

Not Applicable.
